# A treatment recommender clinical decision support system for personalized medicine: method development and proof-of-concept for drug resistant tuberculosis

**DOI:** 10.1186/s12911-022-01790-0

**Published:** 2022-03-02

**Authors:** Lennert Verboven, Toon Calders, Steven Callens, John Black, Gary Maartens, Kelly E. Dooley, Samantha Potgieter, Robin M. Warren, Kris Laukens, Annelies Van Rie

**Affiliations:** 1 Torch Consortium FAMPOP Faculty of Medicine and Health Sciences, University of Antwerp, Antwerp, Belgium; 2 ADReM Data Lab, Department of Computer Science, University of Antwerp, Antwerp, Belgium; 3 Department of Internal Medicine and Infectious Diseases, Ghent University Hospital, Ghent, Belgium; 4 Department of Internal Medicine, University of Cape Town and Livingstone Hospital, Port Elizabeth, South Africa; 5 Division of Clinical Pharmacology, Department of Medicine, University of Cape Town, Cape Town, South Africa; 6 Divisions of Clinical Pharmacology and Infectious Diseases, Johns Hopkins University School of Medicine, Baltimore, MD USA; 7 Division of Infectious Diseases, Department of Internal Medicine, Faculty of Health Sciences, University of the Free State, Bloemfontein, South Africa; 8 Division of Molecular Biology and Human Genetics, DSI-NRF Centre of Excellence for Biomedical Tuberculosis Research, SAMRC Centre for Tuberculosis Research, Stellenbosch University, Cape Town, South Africa

**Keywords:** Clinical decision support system, Treatment individualisation, Machine learning

## Abstract

**Background:**

Personalized medicine tailors care based on the patient’s or pathogen’s genotypic and phenotypic characteristics. An automated Clinical Decision Support System (CDSS) could help translate the genotypic and phenotypic characteristics into optimal treatment and thus facilitate implementation of individualized treatment by less experienced physicians.

**Methods:**

We developed a hybrid knowledge- and data-driven treatment recommender CDSS. Stakeholders and experts first define the knowledge base by identifying and quantifying drug and regimen features for the prototype model input. In an iterative manner, feedback from experts is harvested to generate model training datasets, machine learning methods are applied to identify complex relations and patterns in the data, and model performance is assessed by estimating the precision at one, mean reciprocal rank and mean average precision. Once the model performance no longer iteratively increases, a validation dataset is used to assess model overfitting.

**Results:**

We applied the novel methodology to develop a treatment recommender CDSS for individualized treatment of drug resistant tuberculosis as a proof of concept. Using input from stakeholders and three rounds of expert feedback on a dataset of 355 patients with 129 unique drug resistance profiles, the model had a 95% precision at 1 indicating that the highest ranked treatment regimen was considered appropriate by the experts in 95% of cases. Use of a validation data set however suggested substantial model overfitting, with a reduction in precision at 1 to 78%.

**Conclusion:**

Our novel and flexible hybrid knowledge- and data-driven treatment recommender CDSS is a first step towards the automation of individualized treatment for personalized medicine. Further research should assess its value in fields other than drug resistant tuberculosis, develop solid statistical approaches to assess model performance, and evaluate their accuracy in real-life clinical settings.

## Introduction

Evidence-based medicine aims to integrate individual clinical experience with the best available external scientific evidence to develop trustworthy clinical practice guidelines and optimize clinical decision making [1, 2]. Under the evidence-based medicine paradigm, the clinician uses sound evidence to formulate the best therapeutic choice for their patient, most often through a standardized public health approach where national or international guidelines are implemented. More recently, increasing attention is given to personalized medicine where medical decisions are tailored to the individual patient based on their predicted response to treatment in order to administer “therapy with the right drug at the right dose in the right patient” [3, 4]. To implement personalized medicine, diagnostic tests are performed to determine the patient’s and/or the pathogen’s phenotypic and genetic characteristics. Integrating individual patient genomic information into a clinical decision is challenging however, especially for non-experts, given the rapid evolution in knowledge on the genotype–phenotype associations. The use of a clinical decision support system (CDSS) could facilitate the use of personalized medicine approaches by less experienced physicians and other health care workers at the time and location of patient care [5, 6].

CDSSs for guiding treatment decisions can either be data-driven or knowledge-driven. Knowledge-driven CDSSs use a rule-based system, implement guidelines developed by national or international organizations such as CDC and WHO, and operate at a rather coarse level and do not consider all available patient or pathogen information [7, 8]. In contrast, data driven CDSSs use techniques such as machine learning and data mining and aim to use all relevant data to learn complex relations and dynamics from past experience and reveal patterns in the data in order to assist with the complex decision making. For personalized medicine, data-driven CDSSs are attractive as data is increasingly being collected and stored [9–13].

Recommender systems use machine learning and data mining techniques to predict the preference a user would give to a specific item based on their preference history [14]. Recommender systems are mostly used to make personalized recommendations in e-commerce (e.g. Amazon), online media (e.g. Netflix), social media, and online news feeds [15]. Most recommender systems either use collaborative filtering, content-based filtering, or a combination of the two. Collaborative filtering recommender systems predict which items a user might like based on other similar users that liked similar items [16] and implicit (i.e. a user watched a movie) or explicit (i.e. a user gave a 5-star rating) preferences by the user. Content-based recommender systems predict which new items the user will like by learning a classifier of the likes and dislikes of a user using the features associated with the items they like. [14, 17]. Data driven CDSSs and recommender systems are uncommon in clinical practice, mostly due the perception that they are a ‘black box’ tool [6] with a decision process that lacks transparency, even though transparent recommender systems exist [18, 19].

Crowdsourcing is a problem-solving model in which a large open group of actors try to collectively solve a larger problem [20] with many applications such as street mapping (OpenStreetMap; a collaborative effort of mappers contributing to create and maintain world map data), and data science (Netflix Prize; an open competition to develop the best collaborative filtering algorithm for Netflix [21]).

In this study we aimed to develop a fully automated hybrid knowledge- and data-driven treatment recommender CDSS, utilising crowdsourcing to train and asses model performance, to identify the optimal treatment regimen for individual patients. In addition, we apply the personalized medicine approach to drug resistant tuberculosis as a use case to explain the novel CDSS method development process and its potential application in global health. Drug resistant tuberculosis was chosen as a proof-of-concept as it is recommended that a patients’ treatment regimen is individualized upon receipt of drug susceptibility testing results. However, due to the lengthy and complex process treatment individualization is not always implemented in routine care [22].

## Methods

**Fig. 1:**
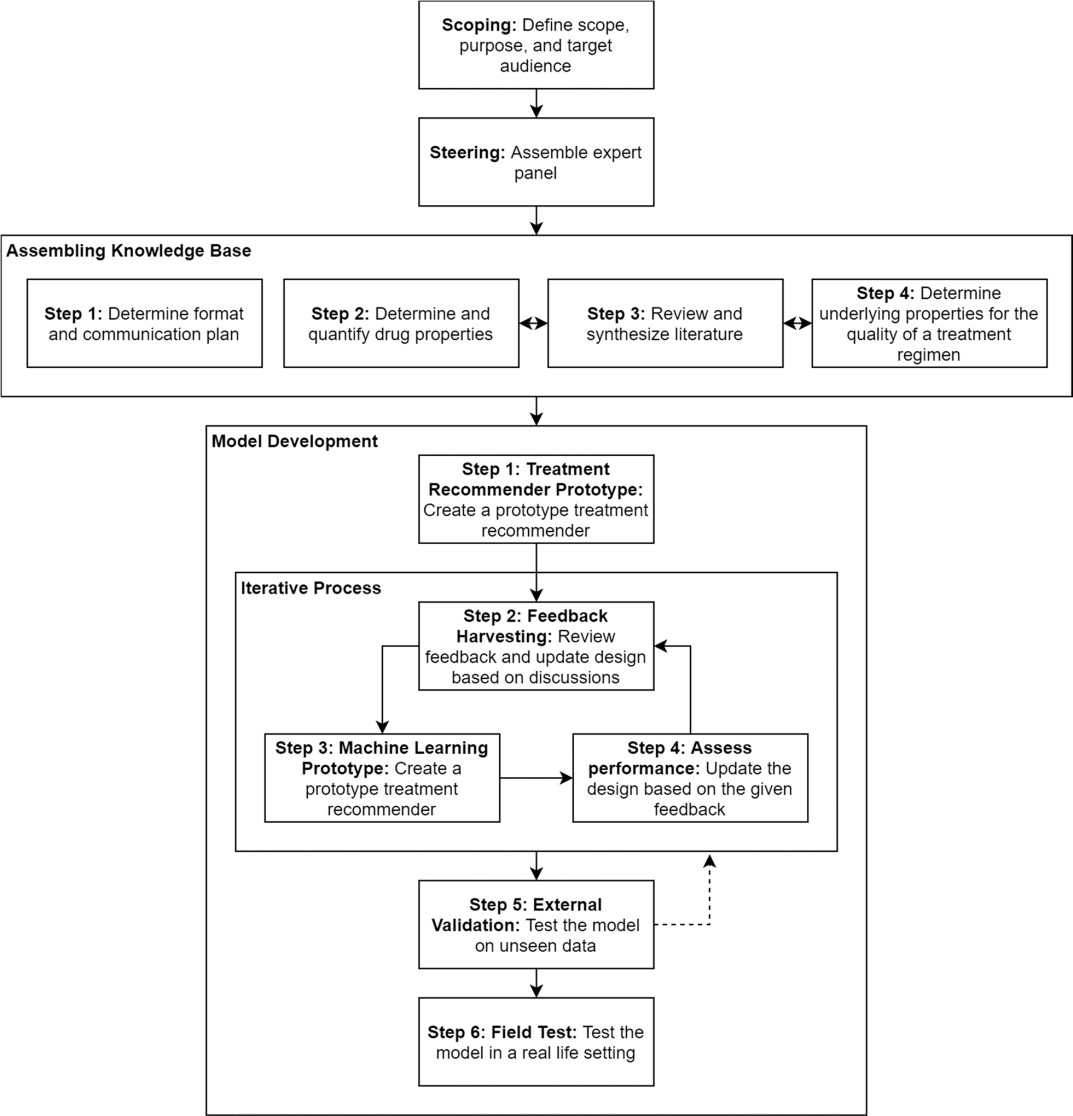
Steps in the development process of an automated clinical treatment recommendation system

To ensure a transparent and standardized process, we adapted the multi-step approach for the development of a decision aid [23] (Fig. 1). This standardized approach provides a framework that can easily be adapted for the development of a CDSS [23]. In this methods section, we describe the complete process of developing the treatment recommender CDSS. In the results section, we present the results of the application of this developed treatment recommender CDSS for the individualized treatment of drug resistant tuberculosis.

### Defining the scope and assembling the expert panel

After defining the purpose and target audience of the treatment recommender CDSS (scoping step), an expert panel was assembled (steering step). To ensure a multidisciplinary and holistic approach, the panel represented expertise in pathogen genomics (knowledge of genotype–phenotype associations), pharmacology (characteristics of the drugs, drug-drug interactions, and synergy and antagonism between drugs), clinical experts, a health informatics technology or computer science expert, as well as health system and health economic experts. In addition, patients were consulted to provide their perspective (e.g. the impact of side effects on their quality of life).

### Assembling the knowledge base

The knowledge base was developed in an iterative manner by combining review of published literature, non-published data when gaps in the literature were identified, and consensus building between experts using a standardized format for efficient expert feedback. First, the key features of relevant individual drugs were determined. Next, the features of the treatment regimen were established, hereafter referred to as regimen features, including the number of effective drugs required and how drug features are aggregated into regimen features. Third, data input requirements and decisions on user-friendly design of the treatment recommender and communication of the recommendation were made.

### Model development

Development of the model consists of 6 steps (Fig. 1). First, a prototype was developed to rank all possibly valid treatment regimens for an individual patient. Second, expert feedback was harvested on a sample of the top scoring regimens for patients that are representative of the target population. Third, the expert feedback was used to develop a training dataset. Fourth, the model performance in recommending the optimal individual treatment regimen was assessed. When the model performance was shown to be suboptimal, the rankings for the patient-regimen pairs obtained by the random forest classifier were used in determining the sampling for the next round of expert feedback harvesting. This process of machine learning, expert data harvesting and assessment of model performance was repeated until the model no longer substantially improved. Fifth, the final model was tested on a different real-life clinical dataset to assess the degree of overfitting to the training data and verify that the model is transferable to new data. Overfitting occurs when a model corresponds too closely or exactly to the data on which it was trained and may therefore underperform on new and unseen data [24]. The final step in the development process is a field test to assess the effectiveness of the treatment recommender CDSS model for individualized treatment in clinical trial participants. In the intervention arm, the minimum required patient information and data on genomics information (treatment recommender CDSS input) is used by the treatment recommender CDSS to proposes the optimal individualized treatment for that patient (treatment recommender CDSS output). In the section below, we describe the first three steps in greater detail.

#### Step 1: Developing the treatment recommender prototype

**Fig. 2:**
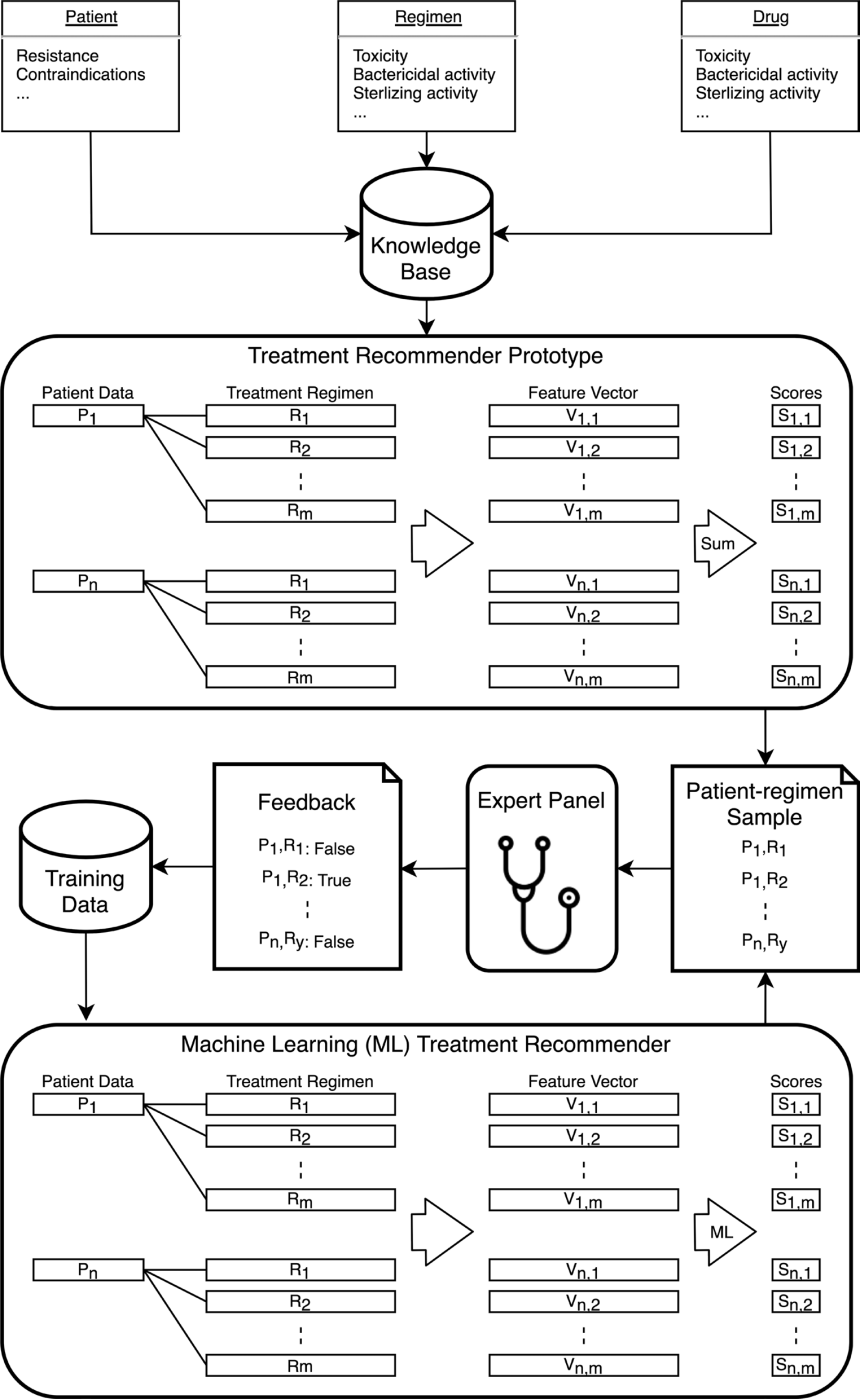
The model development flow

Based on the knowledge base assembled, the prototype computes a quality score for every valid patient-regimen pair (Fig. 2). A valid regimen is defined as a regimen that only contains valid drugs, and valid drugs are defined as drugs that are effective (no resistance detected) and can be included in the individualized treatment regimen because of absence of clinical contraindications, drug stock outs, or country-specific drug licensing issues.

The number of possible regimens *r* for a given patient is:

1 $$\begin{array}{*{20}c} {r = \left( {\begin{array}{*{20}c} n \\ k \\ \end{array} } \right) = \frac{n!}{{k!\left( {n - k} \right)!}}} \\ \end{array}$$

where *n* is the number of drugs available for a specific patient and *k* is the number of effective drugs required in a treatment regimen. If the number of drugs to be included in the individualized treatment regimen can vary, then the total number of possible regimens for each patient equals the sum of formula 1 for all possible values of *k*.

The total number of unique resistance profiles *rp* for a disease is:

2 $$rp = \mathop \sum \limits_{k = 0}^{d} \left( {\begin{array}{*{20}c} d \\ k \\ \end{array} } \right) = 2^{d}$$

where *d* is the total number of available drugs for the disease of interest.

In the prototype, all regimen features were normalized from 0 (bad) to 1 (good) on the patient level, meaning that the entire 0 to 1 interval is used to represent each regimen feature even in patients with few available drugs. A higher score can be interpreted as better for the patient and the highest scoring regimen is assumed to be the best regimen for that patient. After normalizing all regimen features and inverting negative features, such that for all regimen features a lower score means worse for the patient, the sum of all features equates to the quality score for that regimen, which is then used to rank the regimens for individual patients.

#### Step 2: Harvesting expert feedback

For each of the patients selected to represent the target patient population, a sample of the top scoring regimens was reviewed by clinicians experienced in treating the condition of interest (Fig. 2). The number of cases reviewed was fixed and set to be large enough to generate sufficient data to train the machine learning model but small enough so that the experts were able and willing to carefully review every case presented. We sampled with replacement to allow that multiple experts provide feedback on the same patient-regimen pair, to allow that experts can provide feedback on the same patient-regimen pair multiple times, and to ensure that a single expert cannot veto a top scoring regimen. The sampling function randomly samples 1 regimen from the top 3 regimens (based on the ranking) for that patient. A regimen *i* that has been sampled before however has a probability *p*_*i*_

3 $$\begin{array}{*{20}c} {p_{i} = 1 - p_{initial}^{{f_{i} }} } \\ \end{array}$$

where *p*_*initial*_ is the initial probability and *f*_*i*_ is the amount of feedback already received for regimen *i*, to be removed from the top 3 making room for the next regimen in the ranking. Parameter *p*_*initial*_ can be tuned to make regimens more or less likely to be removed from the top 3. After tuning, we found *p*_*initial*_ = 0.2 to give a good balance between resampling a regimen and allowing new, lower ranked regimens to be sampled.

**Fig. 3:**
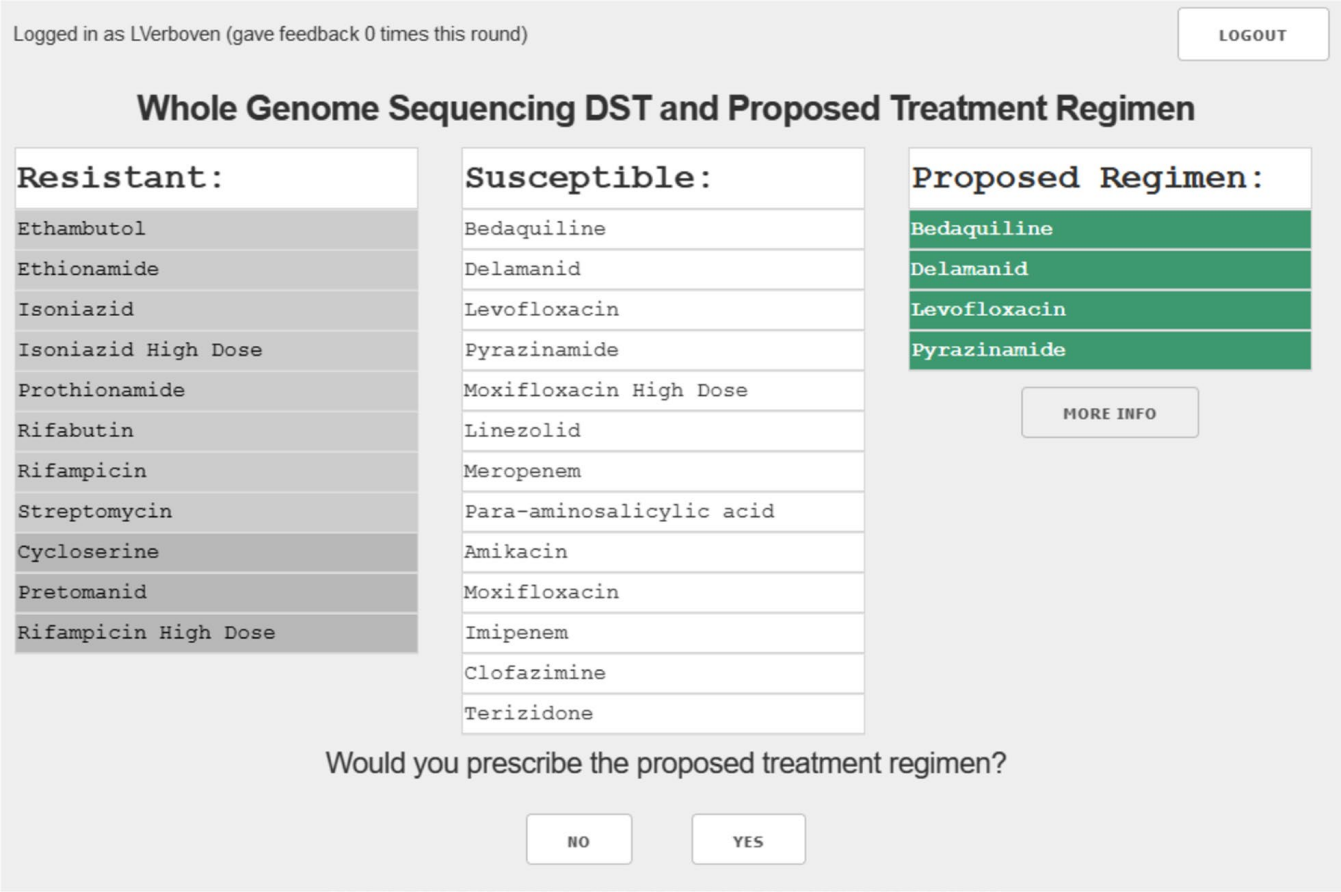
Layout of the web interface

To harvest the experts’ feedback on the regimen-patient pairs, we developed a secure web interface (Fig. 3) that captured the feedback in a structured format. The web interface contained four components: list of drugs that are not valid for this patient (due to resistance, contraindications or not being available), list of valid drugs for the patient, and the recommended treatment regimen. Upon review of this information, the expert is asked whether they would prescribe this regimen for the individual patient. If the expert responded they would not prescribe the recommended regimen to the individual patient, they were asked why (open field) and were asked to list the regimen they would prescribe. The alternative regimen increased the number of patient-regimen pairs with positive feedback. The reasons for rejection were used to identify recurring topics that were then discussed with the experts. The outcome of these discussions led to additional literature review and possible addition, modification, or removal of drug or regimen features.

#### Step 3: Data driven machine learning model development

Most recommender systems are machine learning systems that help users discover new products and services based on the user’s history and preferences. Our recommender model is different from standard recommender systems in that it does not use a patient’s history to propose a new treatment for that patient. Instead, our recommender model learns the underlying qualities of treatment regimens such that a good regimen can be recommended for an individual patient with a specific *Mycobacterium tuberculosis* drug resistance profile. The feedback given by the experts on what was considered a good treatment regimen was stored in a database and served as training data for the machine learning model. We used a random forest classifier to learn the importance of features and interactions between the features of a treatment regimen because random forest classifiers are robust against overfitting and easy to develop. An additional advantage of random forests is that they are constructed by having many decision trees vote and taking the consensus of these trees [25, 26]. The concept of a decision tree and the way they are used in random forests is very intuitive, making these more likely to be accepted by clinicians. Using the normalized features, the model learns which regimen is good for a given patient, accounting for the fact that not all options are available. The model is trained on the patient-regimen-feedback pairs obtained through the expert feedback harvesting step and tries to predict the probability that a patient-regimen pair was deemed good by the experts (Fig. 2). These probabilities are then again used to rank all patient-regimen pairs for a given patient, with the highest ranked regimen being the optimal treatment for that patient.

We used a patient level leave-one-out cross validation strategy to predict the new ranking of regimens for a given patient. In other words, when using the model to predict the ranking for patient *p*, we used the entire training data excluding all training data on *p* to train the random forest classifier.

#### Step 4: Assessing model performance

We used three patient level performance measures: Precision at 1 (P@1) which assesses the highest ranked regimen, Mean Reciprocal Rank (MRR) which represents the average of how high the first regimen is ranked over all patients by the model, and Mean Average Precision (MAP) which takes the position of all appropriate regimens into account. P@k, MRR, and MAP are performance metrics often used in information retrieval [27–29]. Information retrieval (IR) is defined as finding material (documents) that satisfies and information need from within large collections [27]. Our CDSS closely resembles the IR problem in that we try to identify a few -in our case just one, the highest ranked- regimens for a specific resistance profile given the regimen features among a large collection of possible treatment regimens.

P@1 is a performance parameter where precision at N is defined by Eq. 4.

4 $$P@N = \frac{\# good\,treatments\,in\,top\,N}{{\# total\,treatments\,in\,top\,N}}$$

P@1 is the fraction of patients for which the top ranked regimen is classified by the experts as an appropriate regimen, with appropriate regimen defined as a regimen the expert would be willing to prescribe for that patient. When experts disagreed on the highest ranked regimen, a majority voting was used to determine whether the regimen is appropriate.

The mean reciprocal rank is defined by Eq. 5,

5 $$\begin{array}{*{20}c} {MRR = \frac{1}{\left| P \right|}\mathop \sum \limits_{p}^{P} \frac{1}{{{rank}_{p} }}} \\ \end{array}$$

where *P* is the set of patients and *rank*_*p*_ the position of the first regimen classified by the experts as appropriate for patient *p*.

The mean average precision is defined by Eq. 6,

6 $$\begin{array}{*{20}c} {MAP = \frac{1}{\left| P \right|}\mathop \sum \limits_{p}^{P} AvgP\left( p \right)} \\ \end{array}$$

where *AvgP*(*p*) is defined in Eq. 7

7 $$\begin{array}{*{20}c} {AvgP\left( p \right) = \frac{1}{{\left| {{GT}_{p} } \right|}}\mathop \sum \limits_{i}^{{{TX}_{p} }} (P@i \times rel\left( i \right))} \\ \end{array}$$

where *GT*_*p*_ is the set of appropriate treatments for patient *p*, *TX*_*p*_ is the set of all valid treatment regimens for patient *p*, and *rel*(*i*) indicates whether treatment *i* is an appropriate treatment for patient *p* (*rel*(*i*) = 1), or an inappropriate treatment (*rel*(*i*) = 0).

## Results

In this section, we present how the newly developed method was applied as a proof-of-concept to develop a treatment recommender CDSS to guide the individualization of treatment for Rifampicin Resistant Tuberculosis (RR-TB).

The scope and purpose of the development of a RR-TB treatment recommender was defined as “improving RR-TB treatment outcomes by optimizing the individualized treatment regimen in high TB burden resource limited settings”. The primary target audience were the clinicians treating patients with RR-TB in such setting.

### Composition of the RR-TB expert panel

A multidisciplinary steering group of experts was assembled by inviting clinicians with experience in treating RR-TB, pharmacologists with expertise in TB drugs, molecular epidemiologists with expertise in interpretation of the genotype–phenotype associations regarding drug resistance in *Mycobacterium tuberculosis*, health systems experts to assess integration of individualized treatment in routine care, and ex-RR-TB patients.

### Feature selection for the RR-TB treatment recommender model

The design phase started with a stakeholder meeting where all the members of the steering group discussed the treatment recommender input parameters. Discussions focussed on number of drugs needed in an effective treatment regimen, health system burden of treatment monitoring, monitoring burden, drug toxicity, drug features, and clinical patient characteristics, and genomic drug resistance profile. Uncertainties regarding the input parameters identified during the meeting were resolved through literature search, identification of unpublished data and iterative discussion until a consensus was reached on all features to be included in the model. These discussions resulted in a set of 24 drugs that are licensed for treatment of tuberculosis in South Africa, 9 drugs features, 18 regimen features, and the consensus that 4 effective drugs need to be included in all individualized regimens (Table 1). Based on these decisions, up to 10,626 valid treatment regimens were possible (Eq. 1) for a patient without resistance.

**Table 1:** Knowledge base on drugs, drug features and regimen features included in the treatment recommender for drug resistant tuberculosis

Drugs	Amikacin, Bedaquiline, Clofazimine, Cycloserine, Delamanid, Ethambutol, Ethionamide, Imipenem, high or standard dose Isoniazid, Levofloxacin, Linezolid, Meropenem, high or standard dose Moxifloxacin, Para-aminosalicylic acid, Pretomanid, Prothionamide, Pyrazinamide, Rifabutin, high or standard dose Rifampicin, Streptomycin, Terizidone
Drug features	Route of administration, toxicity, QT prolongation, cost, early bactericidal activity, bactericidal activity, sterilizing activity, mechanism of action, propensity to acquire resistance
Regimen features	Core or companion drug^a^ [30], prevention of acquired resistance^a^, four different mechanisms of action^a^, fully oral regimen^a^, cost^b^, toxicity^b^, QT prolongation^a^, high early bactericidal activity^a^, early bactericidal activity^b^, high early bactericidal activity^a^, bactericidal activity^b^, high sterilizing activity^a^, sterilizing activity^b^, synergism^b^, antagonism^b^, contra-indications^a^, same drug class^a^, first line drugs included^b^

^a^Binary features, ^b^Continuous features

### Training and validation RR-TB datasets

We used a dataset that contained both clinical and whole genome sequencing data on 355 patients diagnosed with RR-TB in three provinces in South Africa. The whole genome sequencing data represented 129 different drug resistance profiles. A group of 6 clinicians experienced in treatment of drug resistant tuberculosis were then asked to provide feedback on the recommended treatment using a structured online survey (Fig. 3).

### Assessment of RR-TB treatment recommender model performance

Assessment of the performance of the prototype model showed P@1 of 89%, mean average precision (MAP) of 53% and mean reciprocal rank (MPR) of 90% (Table 2). The written comments on regimens judged to be inappropriate were discussed with the expert group which resulted in modifications to the treatment recommender CDSS prototype. For example, the efficacy of the different drugs, which was initially quantified using a single feature was changed to three features: early bactericidal activity, bactericidal activity, and sterilizing activity. Using the feedback, a random forest classifier was used to identify complex relations and patterns in the data. Based on these results, the updated treatment recommender CDSS reclassified the order of valid treatment regimens from which a sample was drawn for a second round of feedback harvesting from the expert clinicians. The model had improved, with an increase in all three performance parameters. P@1 increased from 89 to 95%, MAP from 53 to 69% and MRR from 90 to 97%. After three rounds, the performance no longer improved, with P@1 and MRR stabilizing at 95% and MAP around 70%. The increase in P@1 from 89% of all resistance profiles after the first round to 95% after the third round of feedback harvesting shows that the proportion of correct recommendations of an accepted regimen increased. The MRR shows that on average the first accepted regimen is ranked higher after multiple rounds of feedback harvesting with a small decrease after round 3. Combining the change in MMR with the increase of P@1 indicates that where the model still makes mistakes, the mistakes are less severe. Finally, the change in MAP shows that on average all the accepted regimens are ranked higher after three rounds of feedback harvesting, indicating that the model is learning the underlying properties of what constitutes a good treatment regimen.

**Table 2:** Training and external validation of the treatment recommender CDSS model

	Number of regimens presented to experts	Total number of observations^a^	Number of participating experts	P@1 (%)	Mean average precision (%)	Mean reciprocal rank (%)
Training round 1	479	855	5	89	53	90
Training round 2	445	719	6	95	69	97
Training round 3	360	607	6	95	72	95
Training round 1–3	1284	2181				
External validation	375	592	5	78	68	87

^a^Number of observations include the alternative proposed by the expert in case the recommended regimen was not considered appropriate by the expert. The performance figures for the training rounds indicate the performance when training and validation on all currently available training data

For the external validation, another dataset consisting of 64 unique resistance profiles for patients diagnosed with RR-TB in another province of was used. On this external validation set, the model performance was lower, with a P@1 of 78%, MAP of 68% and MRR of 87% (Table 2). Based on the improvement in the metrics over three rounds and after review of the CDSS recommendations that were not considered appropriate by the experts, the expert panel decided that additional rounds of feedback harvesting would most likely not substantially improve the CDSS performance and that the performance was sufficient for evaluation in a research setting.

## Discussion

We developed a novel treatment recommender CDSS that combines a knowledge-driven approach using feedback harvesting methodology with a data-driven approach using machine learning methods to automate the individualisation of treatment. The knowledge-driven component consists of input and feedback from stakeholders and experts. The data-driven component consists of the machine learning methods to identify complex relations and patterns.

Our approach is fundamentally different from standard knowledge-driven approaches to CDSSs and offers several advantages for personalized medicine. Standard knowledge-driven systems implement clinical guidelines using if–then rules which allow limited treatment individualisation [6] and offer little flexibility as they need a complete overhaul when new drugs become available or new knowledge becomes available. In contrast, including new drugs and incremental knowledge on existing drugs in our novel methodology is possible without new model training through quantifying the features of the new drug or updating the relevant drug features.

Our approach is also fundamentally different from the data-driven methods that have been used for other CDSSs. Grasser et al. developed two therapy decision support systems that use historical treatment data to individualise psoriasis treatment based on patient attributes and past treatment attributes to predict the response to different therapies [6]. The main limitations of this methodology are that it does not learn the underlying properties of optimal treatment but bases decisions on which treatments have worked well in the past similar patients. Consequently, this method suffers from concept drift where statistical properties of data change over time due to the discovery of a new drug or new knowledge on drug features. Our model is less subject to concept drift, as it is possible to assign features to a new drug or update the features of the drugs when statistical properties of drug or regimen features change.

Machine learning and artificial intelligence methods such as neural networks have been used in the framework of personalized medicine to learn complex and nonlinear relationships between prognostic features and an individual patient’s risk of treatment failure [11]. This approach requires a dataset with treatment outcomes. Because of the relative novelty of personalized medicine, such datasets with treatment outcome only allow the model to learn the underlying properties of the current standard of care instead of novel individualized treatment regimens.

The application of our newly developed treatment CDSS methodology to the individualization of treatment of RR-TB provided proof-of-principle by demonstrating that the novel approach is well suited to guide a personalized medicine approach when multiple combinations of drugs are possible in one individual patient. Using the proposed method, a treatment recommender CDSS for personalized treatment can be built in a relatively short period of time using a combination of stakeholder input, published and unpublished evidence, and expert feedback.

By harvesting expert feedback on patient scenario’s simulated from real-life data, we created a minimal dataset consisting of diverse individual treatment regimens that are representative for patient care. By allowing the experts to provide an alternative regimen when disagreeing with the proposed regimen, the resulting dataset contained a wide variety of accepted and rejected treatment regimens.

While our novel method has many strengths for the field of precision medicine, including the hybrid data- and knowledge-driven approach, the use of a structured ‘crowdsourcing’ approach with predetermined experts for treatment decision making research [31], and the high degree of flexibility, several limitations should be noted. A first limitation relates to the assessment of the model performance. Given the novelty of our approach, there is no consensus method yet to assess the performance of the model and there are no clear decision boundaries on when to stop the iterative development process. The external validation of our model for drug resistant TB demonstrated substantial overfitting on the training data. There are several possible causes for the observed overfitting. Two drugs (pretomanid and high dose rifampicin) were excluded from the validation model because they are not registered for clinical care. The model might have learned some intricate relations between regimens that do and do not include those drugs. Furthermore, the external validation set only included clinical drug resistance profiles not yet seen in the training dataset. These resistance profiles are thus likely less prevalent, resulting in less experience by the experts in treating patients with these drug resistance profiles. The decrease in performance could however also suggest that the current performance parameters may not accurately capture the model’s performance. Using a more diverse set of patient profiles and repeating the development process on this new dataset in combination with the already collected data is likely to reduce overfitting. As such the data now used for external validation could be included as training data before bringing this model into clinical practice. A second limitation is that the model was developed using a limited group of experts. It is unknown to which degree the model development is dependent of the number and composition of the expert group.

While our methodology shows promise to aid clinicians in prescribing the optimal treatment regimen for a patient, several steps are required to implement a treatment recommender CDSS in a clinical setting. Use of other performance parameters and evaluation of the model in clinical trials in different regions of the world will be needed to determine its accuracy for real-life decision making in a personalized medicine. Second several safeguards should be put in place as the treatment recommender CDSS should always be viewed as a tool to aid decision-making and not a substitute for clinical expertise and judgement. The health care worker should thus always review the proposed regimens before prescribing the regimen to their patient. Lastly, the treatment recommender CDSS ideally has an easy-to-use interface that allows communication of the treatment recommendation to health care workers via a secure mobile phone app.

In conclusion, while novel and promising, our hybrid knowledge- and data-driven treatment recommender CDSS for individualising treatment is an important first step in the development of methods aimed to facilitate the widespread implementation of personalized medicine. Further research should assess its value in fields other than drug resistant tuberculosis, develop solid statistical approaches to assess model approaches, and evaluate their accuracy in real-life clinical settings.

## Data Availability

The datasets analysed are available from the corresponding author on reasonable request.

## Abbreviations

CDSS: Clinical Decision Support System
MAP: Mean average precision
MRR: Mean reciprocal rank
P@1: Precision at 1
RR-TB: Rifampicin resistant tuberculosis
